# Imaging and pathological findings of intramedullary inflammatory pseudotumour in a miniature dachshund: a case report

**DOI:** 10.1186/s12917-019-2213-1

**Published:** 2019-12-19

**Authors:** Masamichi Yamashita, Tomohiro Osaki, Yusuke Murahata, Yuji Sunden, Rinko Morita, Tomohiro Imagawa, Yoshiharu Okamoto

**Affiliations:** 1 Joint Department of Veterinary Clinical Medicine, Faculty of Agriculture, Tottori University, 4-101 Koyama-cho Minami, Tottori, Tottori 680-8553 Japan; 2 Ai Doubutsu Clinic, 4-22-28 Higashifukuhara, Yonago, Tottori 683-0802 Japan

**Keywords:** Canine tumour, Spinal cord parenchyma, Paresis, Pseudotumour

## Abstract

**Background:**

Inflammatory pseudotumours (IPTs) are distinctive lesions consisting of myofibroblastic spindle cells and a variety of inflammatory cells. The aetiology of IPTs is unknown. Reports of IPTs in veterinary medicine have been scarse. Moreover, only one case of intradural extramedullary IPT has been previously reported. In this report, we introduce the first known case of canine IPT, which occurred in the parenchyma of the spinal cord.

**Case presentation:**

A 10-year-old female Miniature Dachshund presented with a 2-month-long history of progressively worsening ataxia and tetraparesis. Neurological examination was consistent with a lesion involving the cervical spinal cord. Magnetic resonance imaging revealed an intradural space-occupying lesion in the region of the fourth cervical vertebra. Dorsal laminectomy and resection of the mass were performed. Histopathological examination revealed the proliferation of immature spindle cells (fibroblasts/myofibroblasts and glial cells) and a highly cellular mixture of neutrophils, macrophages and lymphocytic cells. The mass was located in the parenchyma of the spinal cord and was diagnosed as an IPT occurring in the parenchyma of the spinal cord. No causative pathogen was detected. The dog’s symptoms improved, during the first month after surgery. However, neurological symptoms, such as laboured breathing and dysuria, subsequently worsened and the dog died 42 days after surgery.

**Conclusions:**

The present study describes a canine case of IPT occurring in the parenchyma of the spinal cord. The diagnosis and determination of the site of the mass was difficult solely based on preoperative imaging in the present case. The outcome of this case was poorer than that observed in cases of canine extramedullary IPT and human intramedullary IPT, in which the patients exhibited recovery. The prognosis after surgical resection cannot be decided from the present case alone. However, patients should be monitored for potential serious complications and recurrence.

## Background

Inflammatory pseudotumours (IPTs) are distinctive lesions, composed of myofibroblastic spindle cells accompanied by an infiltration of inflammatory cells such as plasma cells, lymphocytes, and eosinophils [1, 2]. The aetiology of IPTs, which can occur throughout the body, is unknown [1, 3].

IPTs are very rare, and reports of their occurrences are limited in veterinary medicine [4–8]. Notably, only one case of intradural extramedullary IPT has been reported [2]. There have been some reports of intra- and extramedullary spinal IPTs in humans [9–12]. The prognosis after surgical resection is favourable for spinal IPTs. A lesion was located from C2 to T10 in a case of human intramedullary IPT, and the outcome was good after complete resection [9]. A case of a canine intradural extramedullary IPT lesion at T13/L1 exhibited a good outcome after resection [2].

In this report, we describe the first known canine case of an IPT occurring in the parenchyma of the spinal cord.

## Case presentation

A 10-year-old female Miniature Dachshund weighing 6.46 kg presented with a 2-month history of progressive thoracic and pelvic limb paresis. The dog underwent three surgical procedures to reposition an inguinal hernia, resect a mammary gland tumour and spay the animal, and resect a mass that was on the surface of the face and not diagnosed by pathological examination by ablation. Administration of prednisolone 1 mg/kg (Nippon Zenyaku Kogyo) daily and stabilization of the neck with a neck collar temporarily resolved the animal’s ataxia, although the paresis continued to progress.

The neurological exam revealed a decrease in the right thoracic limb postural reaction and normal or slightly slow left thoracic limb and bilateral pelvic limb postural reactions. Right thoracic limb spinal reflexes were absent, and left thoracic limb spinal reflexes were decreased. Pelvic limb spinal reflexes were present or exaggerated. Neck pain was also identified without palpation.

**Fig. 1:**
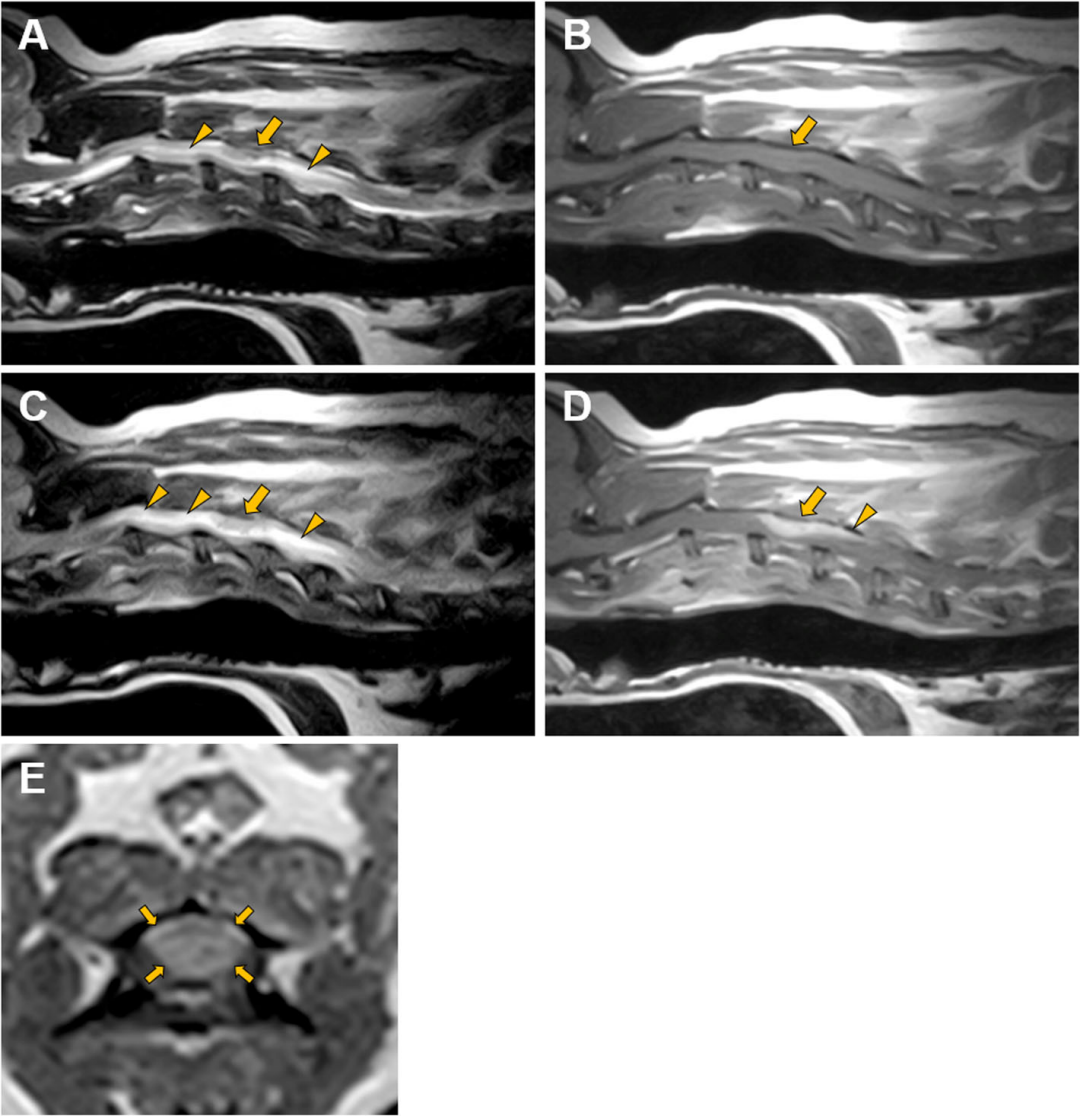
Magnetic resonance imaging of the intradural mass lesion. Sagittal T2-weighted images reveal a mass in the dorsal spinal cord at the fourth cervical (C4) level (yellow arrow) and a large hyperintense lesion from C2 to C6 (yellow arrowheads) (**a**). The mass is isointense (yellow arrow), and the area around the mass is isointense (**b**) on T1-weighted images. The mass is hypo- to isointense (yellow arrow), and the area around the mass is hyperintense on fluid attenuated inversion recovery imaging (yellow arrowheads) (**c**). The mass is homogenously enhanced (yellow arrow) after contrast administration and shows a dural tail sign (yellow arrow head) on T1-weighted imaging (**d**). The mass (yellow arrows) is spherical and homogenously enhanced after contrast administration on T1-weighted transverse imaging (**e**)

A complete blood cell count and serum biochemistry revealed mild increase in serum alanine aminotransferase, a moderate increase in serum alkaline phosphatase, and a mild increase in serum γ-glutamyl transferase. Magnetic resonance (MR) imaging (0.3-T AIRIS Vento, HITACHI, Inc.; Tokyo, Japan) revealed a mass in the dorsal spinal cord at the fourth cervical vertebral (C4) level and a large, hyperintense T2-weighted imaging (WI) lesion from C2 to C6. The mass was hypointense on T2-WI, isointense on T1-WI, hypo- to isointense on fluid attenuated inversion recovery imaging, and strongly and homogenously enhanced on T1-WI postcontrast imaging following intravenous administration of a paramagnetic contrast agent (gadoteric acid; Magnevist, Bayer Yakuhin Ltd., Japan) (Fig. 1). Moreover, postcontrast T1-WI imaging of the lesion showed a dural tail sign.

**Fig. 2:**
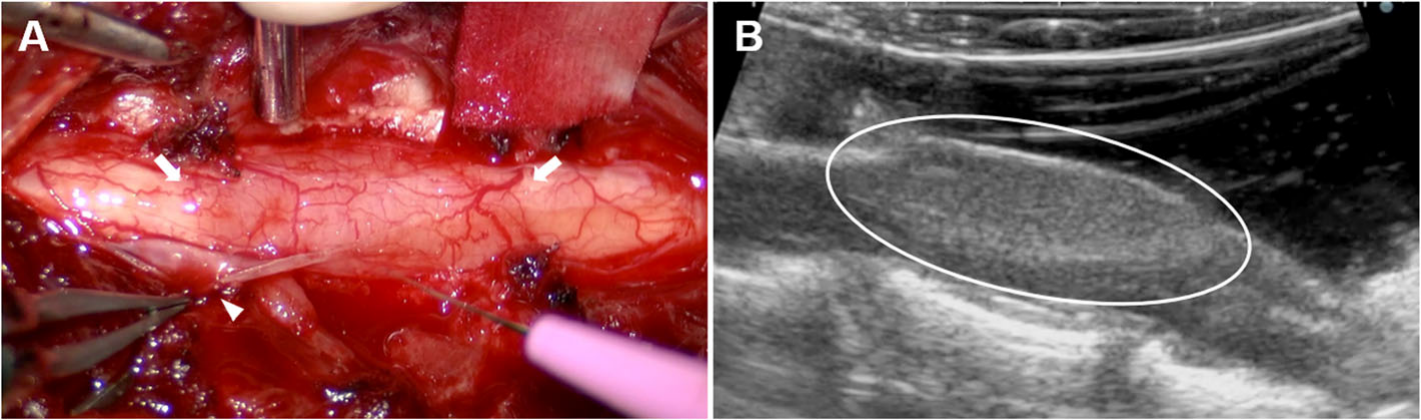
Intraoperative image of the lesion. The arrowhead indicated the incised dura mater. The dura mater covering the mass is thinner than normal dura mater, and the mass is located within the dura mater. The mass is red, indicating angiogenesis. The boundary between the mass and normal spinal cord tissue is unclear (arrow) (**a**). Intraoperative ultrasonic inspection shows that the mass is homogenous and hyperintense relative to the normal spinal cord; the boundary between the mass and normal spinal cord can be confirmed (**b**, circle)

A C3 to C5 dorsal laminectomy, durotomy, and mass resection were performed under a surgical microscope. Intraoperatively, the mass appeared to be located inside the dura mater (Fig. 2a). The surrounding cerebrospinal fluid was transparent. The mass was reddish, angiogenic, and slightly harder than the surrounding normal spinal cord tissue. Intraoperative ultrasonic inspection was thus used to reveal the boundaries of the mass (Fig. 2b). The mass was clearly demarcated and slightly hyperechogenic compared with the normal spinal cord. However, the boundaries between the mass and the healthy spinal cord were unclear, when viewed with the surgical microscope. The body of the mass was easily separated from the normal spinal cord, but the cranial and caudal portions of the mass, which had exhibited the dural tail sign in MR imaging, had poor margins.

After recovery from anaesthesia, the animal exhibited laboured breathing and decreased oxygen saturation for 36 h post-surgery. The dyspnoea was caused by damage to C5-C6, which comprises the phrenic nerve roots and were affected by the surgery [13–15]. A daily dose of prednisolone 1 mg/kg was prescribed after surgery. The animal’s neck pain had apparently improved, and the neurological signs remained unchanged until 1 month postoperatively, at which point the neck pain recurred; laboured breathing and dysuria were also observed at this time. Upper motor neuron paresis was diagnosed as the cause of the dysuria, and the dog could not urinate by itself. Urination was possible by applying pressure to the bladder, which was painful. The laboured breathing gradually worsened. Finally, the dog died at home 42 days postoperatively. The cause of death was thought to be respiratory failure. However, this could not be confirmed because the owner did not permit post-mortem examination.

**Fig. 3:**
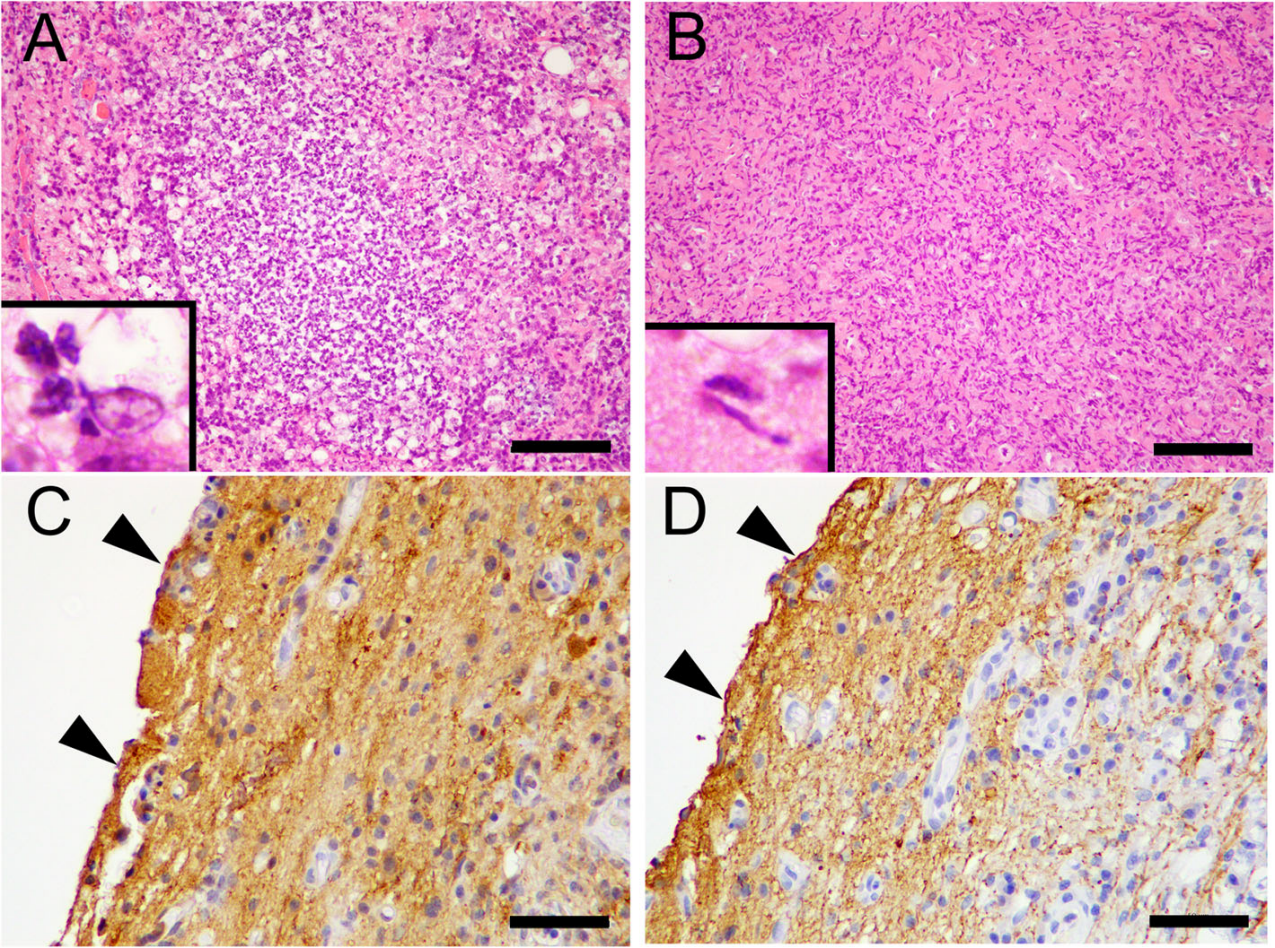
Histopathological images of the inflammatory pseudotumour. These images reveal a highly cellular mixture of neutrophils, macrophages, and lymphocytes (**a**; inset shows the nuclei of macrophages and neutrophils) and numerous chromatin-rich, spindle-shaped cells (**b**, elongated nucleus of activated glial cells). In addition, immunohistochemically- labelled S-100 (nerve cell marker)- and GFAP (astrocytic cell marker)-positive cells are observed within the mass (**c** and **d**). The pia mater (arrowheads in C and D) is observed on the surface of the mass. Scale bars are 100 μm in A and B and 50 μm in **c** and **d**

**Fig. 4:**
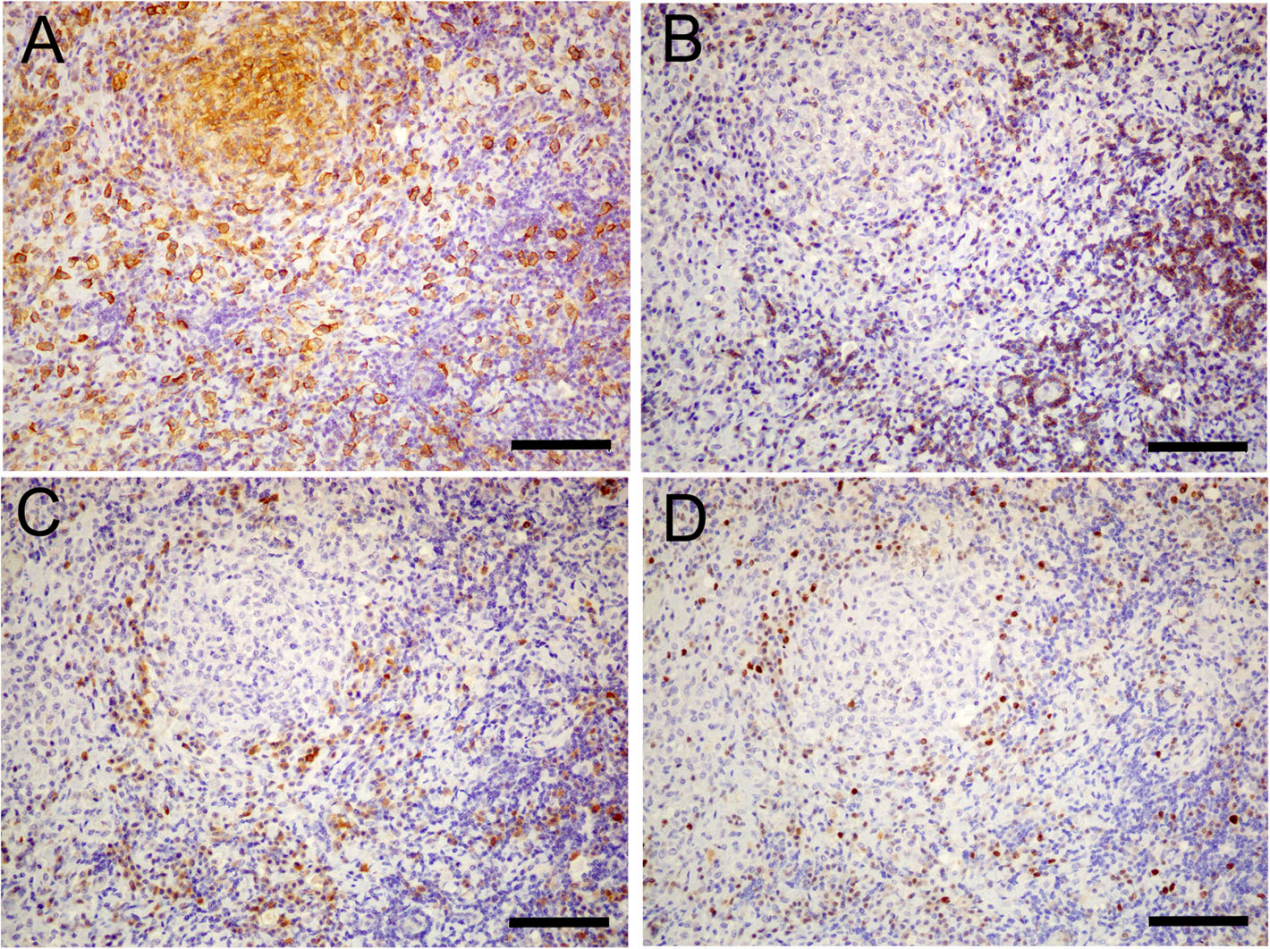
Immunohistological images of immune cells and a cell proliferation marker in mass tissue. Large amounts of Iba-1 (macrophage marker)-positive cells are observed (**a**); CD3 (T lymphocyte marker)- and CD79α (B lymphocyte marker)-positive cells are present (**b** and **c**), as are Ki-67 (cell proliferation marker)-positive cells (**d**). Scale bars are 100 μm

Histopathologic examination of the mass revealed glial cells with infiltration of neutrophils, macrophages, lymphocytes (Fig. 3a and b). The surgical margin of the excised mass was intralesional such that mixed inflammatory cells were located at the periphery of the mass. The mass was located deep in the pia mater, and S-100- (a marker for neuroectodermal origin) and GFAP (a marker for astrocytes)-positive cells were observed in the mass by immunohistochemistry (IHC) (Fig. 3c and d), suggesting that the lesion was located within the spinal cord. Furthermore, the surface of the lesion was covered by pia mater. However, most of the pre-existing spinal tissue, including the pia mater, showed signs of inflammation and some haemorrhage (Fig. 3c and d). Moreover, numerous Iba-1-positive cells (macrophages/microglia) and CD3- and CD79α-positive cells (T and B lymphocytes, respectively) were observed on IHC (Fig. 4). These cells were positive for Ki-67, a proliferative cell marker (Fig. 4d). Periodic acid-Schiff and Gram and Grocott staining were used to detect the presence of pathogens within the lesion. However, no pathogens were found.

## Discussion and conclusions

In the case described here, imaging and surgery were carried out under the assumption that the patient’s space-occupying lesion was a meningioma. However, the lesion was located within the dura mater, as confirmed by intraoperative macroscopic, microscopic, and ultrasonographic examination. Histological findings of the resected mass revealed infiltration of inflammatory cells and a highly immunoreactive cellular mixture of neutrophils, macrophages, and lymphocytic cells. Thus, the first diagnosis, meningioma, was discarded after evaluating the intraoperative findings. The mass located within the dura mater was ultimately diagnosed as an IPT of the parenchyma of the spinal cord by histopathological and immunohistochemical findings. The lesion was diagnosed as an extramedullary meningioma before surgery because the dural tail sign was observed on MR imaging; this sign is regarded as a common and useful marker to distinguish meningiomas from other intracranial and spinal lesions [16–18]. However, it has been reported that other tumours, such as glioblastoma, also exhibit the dural tail sign [16].

A clear definition of IPT with regard to diagnostic criteria is not yet available, especially in the veterinary field. However, the clinical and histopathological findings in the present case are consistent with previous reports of IPT. In particular, the following points support the possibility of IPT: 1) mixed inflammatory cells mainly consisting of lymphocytes and plasma cells and 2) proliferation of spindle shaped cells (fibroblasts/myofibroblasts). Empyema, another inflammatory lesion, is an important differential diagnosis. In empyema, the infiltration of neutrophils is a predominant feature, and the condition is often associated with systemic infections, foreign bodies, or trauma [19, 20]. However, these findings were not observed in the present case.

The MR imaging findings in this case were very similar to those of an earlier intradural extramedullary IPT report [2]. Although we initially presumed that the lesion was extramedullary, it was found within the parenchyma of the spinal cord on histological examination. Since the imaging findings did not differ between the present lesion and previous extramedullary lesions, distinguishing between them was difficult. Thus, it may be necessary to perform a laminectomy, intraoperative ultrasonographic inspection, and histopathological examination to confirm the location of the lesion.

Some researchers have speculated about the aetiology of IPTs. Possible aetiologies include a low-grade inflammatory fibrosarcoma, inflammation following a minor trauma or surgery, an immune-autoimmune mechanism, and pathogenic infection [8, 21–25]. Neutrophilic infiltration was remarkable in the histopathological sections in this patient. However, no pathogens or foreign bodies were observed in the excised mass, and the surrounding cerebrospinal fluid was macroscopically transparent. Given these findings, infections and/or trauma were probably not the cause of the IPT. The cause of the IPT in the present case could not be clearly determined. However, Dachshunds have a predisposition to autoimmune reactive diseases such as sterile panniculitis and other adverse events following vaccine administration; thus, the characteristics of the breed could have been related to the occurrence of the IPT in this case [26, 27].

The outcome in the present case was not satisfactory, especially when compared to a previous extramedullary IPT, in which a dog exhibited stable neurological signs 2.5 years after initial presentation [2]. Moreover, the outcome of the present case was poorer than that of a case of human intramedullary IPT [2]. Cervical dorsal laminectomy has several complications, including neurological deterioration [28]. Signs of laboured breathing were found after surgery in the present case. We thought that the symptom was a complication of the surgery and was indicative of damage to the spinal cord. Moreover, the phrenic nerve roots were locaed at the surgical approach area (C3-C5) in this patient [13, 14]. Injury to the phrenic nerve causes dyspnoea [15], which can be lethal. In this case, the mass invaded the nerve tissue, which led to greater loss of nerve function, than that encountered in other cases of extramedullary IPT. Preoperative nerve damage due to IPT invasion probably increased the severity of surgical complications. We suspect that respiratory failure following laboured breathing was the cause of death, but the source of this recurrence is unclear.

The determination of the location of intra- or extramedullary lesions is crucial for predicting the prognosis of canine spinal cord IPTs. However, MR imaging is often insufficient for distinguishing between intra- and extramedullary lesions. Additional spinal cord IPT investigations are thus required, and exploratory laminectomies may be necessary to diagnose and accurately determine the patient’s prognosis.

## Data Availability

The data are not available for public access because of patient privacy concerns, but are available from the corresponding author on reasonable request.

## Abbreviations

IHC: Immunohistochemistry
IPT: Inflammatory pseudotumour
MR: Magnetic resonance
WI: Weighted imaging
